# Constitution and Legal Basis of Health Rights: Lessons from Cancer Survivors in South Korea

**DOI:** 10.31557/APJCP.2020.21.10.2823

**Published:** 2020-10

**Authors:** Minsoo Jung

**Affiliations:** *Department of Health Science, Dongduk Women’s University, 23-1 Wolgok-dong, Seongbuk-gu, Seoul, South Korea.*

**Keywords:** Health rights, constitution, cancer survivors, Korea

## Abstract

Social fundamental rights can be called ‘factual claims’ of the nationality of individuals, which means rights that require active action by the state for the realization of rights. On the other hand, the ‘normative benefit right’ is the right of the state to demand the enactment of certain laws and regulations. Specifically, the factor to be considered in the analysis of the social rights aspect of the right to health is the ‘publicness of healthcare service’. In order for the right to health to function effectively as a social fundamental right, it must reflect the nature of medical care that is most necessary to maintain the right to health. In the end, the right to health is related to all members of the society and has a ‘normality of publicness’ in the sense that it cannot exclude the benefits of some members. The health care sector, which may cause market failure due to what is termed the asymmetry of information, must be strictly observed in consideration of the essential value of health.

*Constitution and Health Rights*

The right to health includes the right not to be passive with the state, working against one’s own health as well as the right actively to demand that the state maintain one’s health. The right to healthcare means that people can receive national protection for their health and life. The right to health is the right of the people to demand that the absence of infringements on a healthy life for themselves and their families by the state and to demand the necessary benefits and care for maintaining the healthy life of the nation. When we understand the right to health in relation to Article 36 (3) of the Constitution, the right to health not only indicates an obligation of the state to protect health but it also has the characteristic of an objective value order as well as a subjective right. The right to health as a subjective public right has the characteristic of being a social fundamental right that can protect the health and hygiene of people and facilities and the environment as is necessary for the nation as an active consideration while also taking a defensive stand against health infringements by public power. ‘Infringements of health rights’ should include not only realistic infringements but also the ‘possibility of an infringement’ in the preliminary stage, that is, a ‘potential risk of an infringement on health’. Therefore, the contents of the right to health include a prohibition on infringements of health, control of the health of the person, the demand for the creation of a healthy living environment, and the inclusion of the requirement to exclude all activities which incur a risk of a health infringement (Nussbaum, 2011; Rawls, 1971).

The right to health as a fundamental right can be divided into the right to health, the right to health care, and rights in health care (Frankfurt, 1987; Dworkin, 1981). First, the ‘right to health’ is the broadest right to health, which means that an individual has the right to be in a physical and mental state of health. The ‘right to health’ in the passive sense means the right to cope equally with environmental threats affecting health, and the ‘right to health’ in the active sense means equal protection of health from the state, which is a basic right for survival. At this time, ‘health’ basically implies not only the enjoyment of simple health care services but also the right not to be damaged by the social environment (Sen, 1980).

Next, ‘right to health care’ refers to rights as an absolute concept of the access to health care resources and the right to equitable access regardless of any individual’s identity or property. Access to health care is a measure of the right to health care, which is divided into potential access and realized access. The former is related to the characteristics of the health care delivery system and is divided into the supply side and demand side. Potential access from the supply side includes the physical equalization of medical personnel and facilities, and potential accessibility in terms of demand is related to enabling factors, predisposing factors, and needs.

Finally, ‘rights in the health care process’ refers to the fundamental right of an individual to enjoy medical care equally within the health care delivery system. ‘Rights in health care process’ should be understood as having two aspects. First, ‘rights in the health care process’ refers to the right to receive the necessary amount of essential services. Essentially, a necessary amount of a service implies that a service that is deemed fair, with such a judgment usually performed by medical staff, but the understanding of the patient is a prerequisite. Second, ‘rights in the health care process’ means the right to exclude differences at the level of care in equal cases; it is also related to the principle of ‘similar treatment for similar cases’.

If we recognize and secure health rights as specific basic rights, it is difficult to build a foundation on which to combine medical services such as financial resources and delivery systems with market principles. If we want to secure health rights, we must be able to realize this regardless of the financial resources of the nation. The core content of the right to health is ‘minimal health’ along with the most basic elements for securing rights, and it must be developed in accordance with the economic and social situation of the country.

Constitutional scholars have focused on the nature of the fundamental rights of the right to health and the nature of the social right. The right not to be invaded by the state from the viewpoint of the right to freedom and the right to demand that the state should take active consideration of the promotion of the health of the people from the viewpoint of social rights are both linked to this. Health rights have a liberal right and a social right. The basic right of social rights is essentially a structure that cannot be resolved by the norm of the state itself. In other words, even if the basic right of social rights is normatively well established, unless the financial and social basis of the state capable of practicing the norms that have been restored is established, the social fundamental rights remain as a declaration that cannot be understood realistically to exist.

On the other hand, the fundamental right to freedom can be realized only by the fundamental right itself without any other institutional apparatus, and its contents and effects are relatively clear. The right of exclusion of health infringement acts as a liberty right of the right to health is the right to remove the effect of the act on the state when the health of the people is violated by the actions of the state or local governments. Country as defined above includes all national institutions such as the legislative, administrative and judicial branches, but it is the executive branch that enacts the law as the state institution that can generate the most problems in relation to the constitutional right to health. Today, it is difficult to find examples of enacted laws that directly infringe on people’s right to health or on exercising their power for this purpose.

*Health Rights in Terms of Social Rights*

Article 36 (3) of our Constitution emphasizes the obligation to protect the national health of the nation by stipulating that “all citizens are protected by the state in relation to health.” This means that the right to health as a social fundamental right is the most important aspect of health rights. The social right is an individual’s right to demand from the state a de facto benefit, such as a good or opportunity that can be obtained from a person if the person has sufficient financial resources and the supply in the market is sufficient. The social fundamental right is a basic right that guarantees the possibility of ‘opportunity of opportunity’ or ‘possibility of possibility’, unlike the basic liberty right which provides the opportunity or possibility of freedom, that is, an inviolable space. It is structurally different from the fundamental freedoms that are understood essentially as the right of defense against the state in terms of the activities of the state supplying specific goods and services, such as material and facility benefits. In addition, because it is directly linked to financial investment, the conditions and methods of concrete realization are essentially different from those pertaining to the fundamental right of the right to liberty.

Social fundamental rights can be called ‘factual claims’ of the nationality of individuals, which means rights that require active action by the state for the realization of rights. On the other hand, the ‘normative benefit right’ is the right of the state to demand the enactment of certain laws and regulations. Specifically, the factor to be considered in the analysis of the social rights aspect of the right to health is the ‘publicness of healthcare service’. In order for the right to health to function effectively as a social fundamental right, it must reflect the nature of medical care that is most necessary to maintain the right to health. In the end, the right to health is related to all members of the society and has a ‘normality of publicness’ in the sense that it cannot exclude the benefits of some members. The health care sector, which may cause market failure due to what is termed the asymmetry of information, must be strictly observed in consideration of the essential value of health.

What the public can actively demand from the nation with regards to the right to health is to provide facilities and an environment for the maintenance of health and to provide appropriate medical services to recover infringed health (Sandel, 1982). In other words, it is the content of the right to health as a social fundamental right to provide the facilities, an environment and medical services necessary for health maintenance, and provide medical services to recover infringed health. Regarding , regular preventive measures of health screening, infectious disease management, natural environment management, conservation of water quality, the management and monitoring of the food distribution process, the promotion of the detoxification of smoking and the prohibition or restriction of tobacco advertising are included. In relation to , the provision of medical services by medical insurance, medical care, emergency treatments, medical institutions established by national or local governments, and the establishment and implementation of comprehensive and systematic health care policy of the state are included. Moreover, the public should be knowledgeable about their health condition and should be able to make their own decisions about the measures necessary to maintain or restore their health. The essential premise for this is the guarantee of access to medical information for the public.

*Remarks*

What is problematic is how the above contents of the right to health as a social fundamental right have a certain degree of effectiveness and normative power. If the right to health as a social fundamental right retains within what has been described as the ‘characteristic of program regulations’ or ‘abstract rights’, the content of the right to health, which has been examined in terms of social fundamental rights, becomes empty. There have been conflicting views on the legal nature of social fundamental rights, as follows (Kymlicka, 2001; Matravers, 2000; Miller, 1976). First, social fundamental rights are not merely concrete rights but are declarations of the social policy goals of the state. Therefore, unless the state enacts the legislation necessary for the realization of its rights, the constitutional provisions thereon cannot claim a juristic claim against the state. In other words, there is a ‘program right theory’ by which judicial remedies cannot be sought through the unconstitutionality of legislation. Second, the national obligation of guaranteeing social fundamental rights is an ‘abstract right’, which is a legal right under the Constitution. Third, the constitutional provisions pertaining to social fundamental rights are those that have direct effects even if there is no legislation to specify them, and those that guarantee social fundamental rights as specific rights. Therefore, there is a ‘concrete right’ theory that states that the state’s omission of the realization of social fundamental rights is a violation of concrete rights and subject to judicial remedies (Tan, 2008).

However, even if they have the same basic social rights, the contents or values they want to guarantee vary. Thus, applying the general theory of fundamental rights to any specific matter can damage the validity of constitutional litigation (Nozick, 1974). It is therefore necessary to distinguish between the different types of social fundamental rights and to personalize them. In order for the right to health as a social fundamental right to be meaningful for constitutional litigation, it is necessary to examine the level of guarantee of the right to health. Therefore, it is necessary to understand the criteria of the Constitutional Court pertaining to the level of the guarantee of social fundamental rights. The Constitutional Court of Korea, as a subjective public institution, minimizes the guarantee of social fundamental rights and judges that the right to claim a definite benefit is granted to the individual. Therefore, if the state does not fulfill the minimum realistic benefit, it can be said that it violates that basic right. In addition, in order to realize social fundamental rights, the state is obliged to guarantee the maximum possible given what is known as the time. Therefore, it is necessary to review the contents of the guarantee of rights broadly and to examine the infringement on the basis of the objectively necessary minimum by lowering the Constitutional Court’s unconstitutional control standard.

According to R. Alexy’s principle model, all social rights grant a subjective right to a prima-facie individual, but this right can only become a definitive right after it is enacted (Anderson, 1999). He classifies the normative structure of social fundamental rights according to the following three criteria. The first is related to whether it is the norm that grants subjective rights to the individual or the norm that imposes an objective obligation on the state. The second pertains to whether it is a binding or non-binding norm. The third is whether the rights and obligations are given definitively or temporarily. First, the view that the right to health as a social fundamental right is not legally binding is the view that the legal nature of social fundamental rights is a program rule. This position entails the guarantee of the right to health as a social fundamental right to the legislator such that the right to health as a social fundamental right is at least legally binding. Next, the right to health as a social fundamental right requires subjective rights. According to the principle of the rule of law, the right should be able to be claimed by the court. In other words, the right to health is a subjective right and should allow criticism of the Constitutional Court or any court when this right is violated. However, if the subjective rights of the right to health are denied and only objective obligations are recognized, the effectiveness of guaranteeing the right to health as a social fundamental right wanes. Article 35 (1) of the Constitution stipulates that “all citizens have the right to live in a healthy and pleasant environment,” and Article 36 stipulates that “all citizens are protected by the state in relation to health.” In other words, our Constitution, which prescribes the right to health, defines the right to health through the form and contents of rights. In the end, according to the Alexy model, health right is a restrictive norm and a definite right as a social fundamental right. However, the extent of guaranteeing and realizing the right to health can vary depending on the social composition, and the difference needs to be viewed from the perspective of the health insurance system.
